# YQFM Alleviates Side Effects Caused by Dasatinib through the ROCK/MLC Pathway in Mice

**DOI:** 10.1155/2020/4646029

**Published:** 2020-08-29

**Authors:** Yuankai Liu, Yujie Dai, Han Xu, Qianliu Zhou, Fang Li, Boyang Yu, Yuanyuan Zhang, Junping Kou

**Affiliations:** Jiangsu Key Laboratory of TCM Evaluation and Translational Research, Research Center for Traceability and Standardization of TCMs, Department of Pharmacology of Chinese Material Medica, School of Traditional Chinese Pharmacy, China Pharmaceutical University, 639 Longmian Road, Nanjing 211198, China

## Abstract

Dasatinib, as a second-generation broad-spectrum tyrosine kinase inhibitor, presents an antitumor effect by inhibiting tyrosine kinases. However, dasatinib causes serious side effects, such as gastrointestinal bleeding and liver toxicity, possibly through the activation of ROCK kinase and MLC phosphorylation. At present, there is no effective prevention and treatment method. Previous research studies have shown that YQFM (YiQiFuMai powder injection) protects the blood-brain barrier by inhibiting the ROCK/MLC signaling pathway; whether YQFM can alleviate the side effects of dasatinib is unknown. In this study, dasatinib was injected (i.p. 70 mg/kg) and YQFM (i.p. 0.336 g/kg, 0.672 g/kg, 1.342 g/kg) was given in advance for 3 days to mice, to explore the effect of YQFM on side effects induced by Dasatinib. The results confirmed that YQFM significantly decreased Evans blue leakage in the small intestine and increased intestinal blood flow, increased the expression of ZO-1, Occludin, and VE-cadherin, and reduced the contents of D-lactic acid, s-VE-cadherin, Alanine aminotransferase (ALT), and Aspartate aminotransferase (AST) in serum. Finally, YQFM inhibited the expression of ROCK-1 and phosphorylation of MLC induced by Dasatinib. These findings suggested that YQFM could improve the side effects caused by Dasatinib linked with the ROCK/MLC signaling pathway, as shown in the graphical abstract.

## 1. Introduction

Among the approved drugs, Dasatinib is the first oral chemotherapeutic agent that can inhibit tyrosine protein kinase in multiple configurations. Dasatinib can inhibit the proliferation of leukemia cells in a chronic myeloid leukemia (CML) bone marrow via various kinases. It is widely used in clinics and has good therapeutic effect. However, Dasatinib has serious side effects [1, 2]. Cardiovascular adverse events (CV-AEs) are considered critical complications in CML patients treated with dasatinib, suggesting probable existance of endothelial damage [3, 4]. The other side effects in clinical reports include gastrointestinal symptoms, bleeding events, pleural effusion [5, 6], pulmonary hypertension [7], hepatotoxicity [8], and renal failure [9]. Some studies have revealed that dasatinib induces the structure of VE-cadherin changes between endothelial cells, increasing the permeability of endothelial cells, leading to blood vessel leakage to induced gastrointestinal bleeding. Dasatinib phosphorylates MLC through the ROCK pathway and induces the aggregation of F-actin structures, which causes the cytoskeleton to remodel and induces hemorrhagic complications [10–12]. On the other hand, when the permeability and integrity of the intestine are destroyed, a large amount of bacterial products enter and damage the liver, increasing the ALT and AST levels in serum [13]. Through investigation of 338 patients taking Dasatinib, it was found that 60% developed liver toxicity [14]. The follow-up study also found that 96 patients had elevated ALT levels and 111 people had elevated AST levels among 186 patients with chronic myeloid leukemia who took Dasatinib for 8 months [15]. Therefore, it is suggested that protecting the connexin by modulating the ROCK/MLC signaling pathway might facilitate to alleviate the side effects caused by Dasatinib.

Sheng-mai San (SMS) is a well-known TCM formula, which included three kinds of medicinal plants: *Panax ginseng* C.A. Mey, *Ophiopogon japonicus* (Thunb.) Ker-Gawl, and *Schisandra chinensis* (Turcz.) Baill. YiQiFuMai powder injection (YQFM) is a modified preparation derived from SMS, which is widely used clinically for the treatment of cardiovascular and cerebral diseases [16–19]. YQFM contains a variety of active ingredients, such as Ginsenoside Rb1, Ginsenoside Rg1, Schisandrin, and Ruscogenin. Ginsenoside Rb1 can be used to treat cerebral ischemia-reperfusion injury and myocardial ischemia-reperfusion injury. Schisandrin A has antiapoptosis and antioxidant effects. Ruscogenin is derived from traditional Chinese medicine, *Ophiopogon japonicus,* and it has anti-inflammatory, antithrombosis, and protective barrier function. It also can prevent the occurrence of hemorrhagic events. The mixture of Rb1, Rg1, and schisandrin can inhibit the caspase-3/ROCK-1/MLC signaling pathway [20].

Modern basic studies have confirmed the beneficial effect of YQFM in animals with cardiovascular or intestinal disease [21–23]. Meanwhile, YQFM also can alleviate stroke, heart failure, and matter-induced acute lung injury and inhibit neuronal apoptosis [24–26]. We previously proved that YQFM could reduce the expression of activated ROCK and inhibit the phosphorylation of MLC, thereby inhibiting the degradation of tight junction proteins and exerting protective effect on the brain microvascular endothelial cell barrier *in vitro* and *in vivo* [27]. However, whether YQFM can protect the intestinal barrier by inhibiting the ROCK/MLC signaling pathway to attenuate the side effects induced by Dasatinib has not been reported.

Therefore, in the present study, we observed the effects of YQFM on intestinal bleeding and liver injury and explored its potential mechanism by measuring the ROCK expression and MLC phosphorylation. To provide some pharmacological evidences for YQFM, that can be a potential application to reduce the side effects induced by Dasatinib.

## 2. Materials and Methods

### 2.1. Materials

YQFM was obtained from Tasly Pharmaceutical Co., Ltd. (Tianjin, China, batch number 20121210). Dasatinib was obtained from CHIA TAI TIAN QING Co., Ltd. (Jiangsu, China, batch number H20133271).

### 2.2. Animals

Male C57BL/6J mice (20–25 g; 8 weeks; specified pathogen-free) were provided by the Model Animal Research Centre of Yangzhou University (Yangzhou, Jiangsu, China) and kept in cages containing standard bedding, with at least five mice per cage. Animal feeding standards strictly follow the standards of “Laboratory Animal Environment and Facilities (GB 14925-2010).” Animals were allowed to acclimatize to their housing environment for at least 7 days prior to experimentation and to the experimental room for 1 h before experiments.

### 2.3. Drug Treatments

YQFM was weighed and dissolved in absolute ethanol to prepare mother liquor. When used, the corresponding concentration was adjusted with physiological saline. Dasatinib was dissolved in physiological saline at the dose of 70 mg/kg, i.p. at 1 h after the administration of YQFM. After 3 days, the mice were sacrificed.

### 2.4. Blood Flow Monitoring

Laser Doppler flowmetry is monitoring of haemodynamics and vasomotor regulation in health and diseases. It is easy be used, and intuitive pictures are obtained [28]. Therefore, laser Doppler flowmetry was selected to monitor blood flow in mice taking Dasatinib, and at the same time, it enriched the clinical examination methods. Thirty minutes before administering Dasatinib, mice were given YQFM, and 2 h later, they were anesthetized with pelltobarbitalum natricum. The small intestine was removed and placed on a Doppler flowmeter for scanning. Images were obtained 1 h later, and the average blood flow was calculated.

### 2.5. Hematoxylin-Eosin Staining

After the animals were sacrificed, the small intestine tissue was placed in an EP tube containing 4% paraformaldehyde and sent to the pathology room of Jiangsu Drug Safety Evaluation Centre for testing.

### 2.6. Evans Blue Leakage Rate Determination

Evans blue (EB) dye has a long history as a biological dye and diagnostic agent. Due to its high water solubility and slow excretion and tight binding to serum albumin, EB is mainly used to assess blood volume and vascular permeability [29]. Therefore Evans blue was used to evaluate the side effects of Dasatinib. Mice in each group were intraperitoneally injected with 70 mg/kg dasatinib on the third day and, then, injected with 5 mg/mL Evans blue solution via the tail vein. After 2 h, the mice were anesthetized. The absorbance of supernatant was measured at 620 nm with a microplate reader, and the content of Evans blue was calculated according to the standard curve.

### 2.7. Determination of ALT and AST and Contents of S-VE-Cadherin and D-Lactic Acid in Mouse Plasma

The plasma is treated according to the manufacturer's guidelines, with a wavelength of 510 nm, measured by using a microplate reader. Plasma was obtained from mice after injecting Dasatinib, with a wavelength of 450 nm, measured by using a microplate reader.

### 2.8. Western Blot Analysis

The proteins from small intestines for western blotting analysis were obtained as previously described [30]. The primary antibodies against ZO-1 (1 : 1000, Abcam, USA), VE-cadherin (1 : 1000, Santa, USA), occludin (1 : 200, Abcam, USA), ROCK-1 (1 : 500, Santa, USA), MLC (1 : 1000, CST, USA), phospho-MLC (1 : 1000, CST, USA), and GADPH (1 : 2000, Bioworld, USA) were used, followed by incubation with peroxidase-conjugated secondary antibodies (1 : 8000, Bioworld, Louis Park, USA). A BCA kit was used to quantify protein and add loading buffer to obtain the western blotting sample, and the loading volume is 30 *μ*g. Bands were demonstrated by enhanced chemiluminescence (ECL).

### 2.9. Statistical Analysis

All results are expressed as the means ± SD. Statistical analysis was performed using Student's two-tailed *t*-test for comparison between two groups and one-way analysis of variance (ANOVA) followed by Dunnett's test when the data involved three or more groups. *P* < 0.05 was considered statistically significant. All analyses were performed using GraphPad Prism Version 5.01 (GraphPad Software Inc. USA).

## 3. Results

### 3.1. Effect of YQFM on Intestinal Blood Flow and Histopathological Changes of the Intestine in Mice with Dasatinib

**Figure 1:**
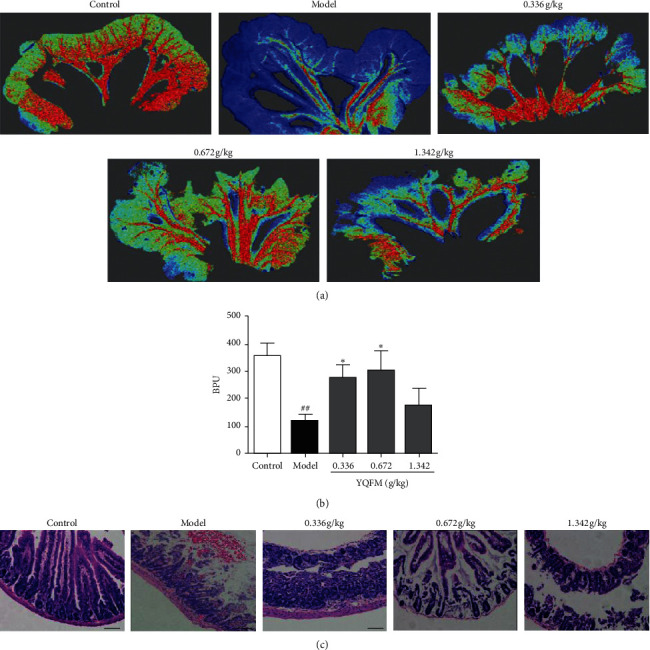
Effect of YQFM on intestinal blood flow and histopathological changes of intestinal sections in mice with Dasatinib. YQFM (0.336, 0.672, 1.342 g/kg, i.p.) was injected intraperitoneally 1 h before being administered with dasatinib (70 mg/kg for 3 d, i.p.). (a) The representative images of intestinal blood flow in different groups. The magnitude of IBF is represented by different colors, with blue to red denoting low to high. (b) Quantitative analysis of IBF in different groups. Data are expressed as the mean ± SD, *n* = 6. ^##^*P* < 0.01 vs. control group; ^*∗*^*P* < 0.05 vs. model group. (c) Hematoxylin-eosin-stained slides of mouse intestinal sections in different groups were examined under a light microscope, and representative stained sections showed that YQFM improves Dasatinib-induced bleeding. Scale bar = 50 *μ*m. *n* = 3.

The literature proves that dasatinib damages the endothelial barrier by destroying the cytoskeletal structure. Laser Doppler flowmetry was used to investigate the effect of YQFM at three different doses on the intestinal blood flow changes caused by continuous administration of 70 mg/kg dasatinib for 3 days in mice. As shown in Figures 1(a) and 1(b), compared with the control group, the blood flow of the small intestine in the model group was significantly reduced (*P* < 0.01). YQFM at two doses of 0.336 and 0.672 g/kg could significantly increase the blood flow of the small intestine caused by dasatinib (*P* < 0.05), indicating that dasatinib could induce bleeding in the small intestine. HE staining was used to further verify the improvement of YQFM on dasatinib-induced small bowel injury. Compared with the control group, the model group experienced significant bleeding, and the structure of the small intestine was destroyed. Compared with the model group, it was found that using YQFM in advance can significantly reduce Dasatinib-induced small bowel barrier destruction and bleeding.

### 3.2. Effect of YQFM on Dasatinib-Induced Intestinal Vascular Leakage and Contents of S-VE-Cadherin and D-Lactic Acid in Mice

**Figure 2:**
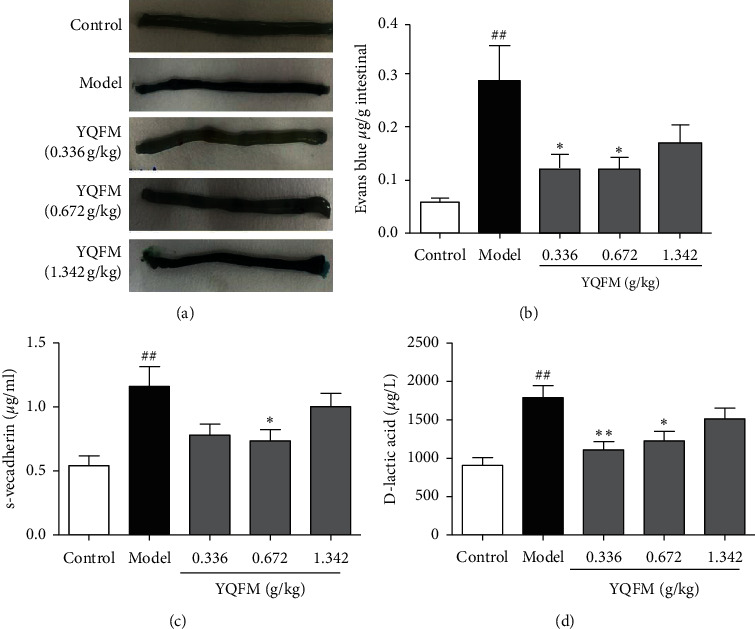
Effect of YQFM on Dasatinib-induced intestinal vascular leakage and contents of S-VE-cadherin and D-lactic acid. YQFM (0.336, 0.672, 1.342 g/kg, i.p.) was injected intraperitoneally 1 h before being administered with dasatinib (70 mg/kg for 3 d, i.p.). (a) Representative gross appearance of the EB-stained intestine of a mouse. Data are expressed as the mean ± SD, *n* = 6. ^##^*P* < 0.01 vs. control group; ^*∗*^*P* < 0.05 vs. model group. (c) Content of S-VE-cadherin was analysed by ELISA. (d) Content of D-lactic acid was analysed by ELISA. Data are expressed as the mean ± SD. ^##^*P* < 0.01 vs. control group; ^*∗*^*P* < 0.05 vs. model mice, ^*∗∗*^*P* < 0.01 vs. model group.

The leakage rate of the Evans blue content is often used to reflect the degree of vascular injury [31]. Therefore, Evans blue was used to investigate the effect of YQFM on small intestinal vascular leakage induced by continuous administration of 70 mg/kg dasatinib for 3 days in mice. The results are shown in Figure 2(a); the intestinal tissue of the model group is darker than that of the control group, indicating that dasatinib could induce vascular leakage. At 0.336 g/kg and 0.672 g/kg, the colour of the intestinal tissue is lighter than that of the model group. As shown in Figure 2(b), the contents of Evans blue in the small intestine tissue of the model group was significantly higher those in the small intestine tissue of the control group (*P* < 0.01), and YQFM at 0.336 g/kg and 0.672 g/kg could reduce the contents of Evans blue (*P* < 0.05). In this section, ELISA kits were used to investigate the effect of YQFM on Dasatinib-induced D-lactic acid and S-VE-cadherin content. As shown in Figures 2(c) and 2(d), the results of ELISA showed that compared with the control group, the serum D-lactic acid and S-VE-cadherin content of the model group were obviously increased (*P* < 0.01) and compared with the model group, YQFM at 0.672 g/kg has significant downregulation contents of D-lactic acid and S-VE-cadherin (*P* < 0.05). These results showed that YQFM plays a role of protect the barrier functions and prevents the degradation of connexins and reduces the intestinal harmful substances in the blood.

### 3.3. Effect of YQFM on the Expression of Connexin

In addition, to evaluate the degree of destruction of the intestinal barrier through Evans blue leakage, we used western bolt to show the degree of destruction of intestinal connexins caused by dasatinib and the protective effect of YQFM. Compared with the control group, the expression of ZO-1, VE-cadherin, and occludin was significantly reduced in the model group. Against this, YQFM at 0.336 g/kg could significantly increase the expression of ZO-1, VE-cadherin, and occludin (*P* < 0.01).

### 3.4. Effect of YQFM on the Contents of ALT and AST in Mice with Dasatinib

Compared with the control group, the ALT and AST contents of the model group increased significantly (*P* < 0.01), and compared with the model group, 0.336 g/kg, 0.672 g/kg YQFM could relieve liver damage and reduce the secretion of ALT and AST (*P* < 0.01).

### 3.5. Effect of YQFM on the ROCK-1/MLC Signaling Pathway in Mice with Dasatinib

Western blot results indicated that dasatinib could significantly increase the expression of ROCK-1 and MLC phosphorylation, which was consistent with the previous reports [11]. Versus to the model group, YQFM could markedly inhibit ROCK-1 activation and phosphorylation of MLC at three doses of 0.336 g/kg, 0.672 g/kg, and 1.342 g/kg (*P* < 0.001).

## 4. Discussion

Vascular endothelial cells are one of the main components of intestinal mucosal tissue. The damage of endothelial barrier can destroy the integrity of the intestinal vascular, increase vascular permeability, and cause vascular leakage. In the tract, the vascular endothelial layer has barrier properties, strictly regulating vascular permeability [32]. The function of endothelial cells in the intestine is gradually being valued. Intestinal vascular endothelial cells are important part of the intestinal mucosa. Different connexins are the key factors to maintain the stability of endothelial cells and maintain the permeability of the intestine [33, 34].

Previous research found that YQFM inhibits the phosphorylation of MLC by reducing the expression of ROCK in bEnd.3 cells in the oxygen-glucose deprivation (OGD) model. In the early stage of OGD (4 h), YQFM can inhibit the combination of MLC and F-actin. Over time (9 h), YQFM improved the degradation of tight junction proteins ZO-1, occludin, and claudin-5 [27]. However, the protective effect of YQFM on the destruction of the intestinal barrier caused by Dasatinib is unknown. In this paper, we infer that YQFM also plays a role in protecting the intestinal barrier in the similar way.

The experiment was designed according to the literature reported [35]. As shown in Figure 1, the blood flow of the small intestine decreased significantly after Dasatinib was injected in the model group, and some studies have shown that the reduction of blood flow is closely related to bleeding [36, 37]. Laser Doppler flowmetry is easy to perform. It can be used to obtain intuitive images and monitor the blood flow of multiple organs and tissues. Laser Doppler flowmetry's result showed that, after injection of Dasatinib, intestinal bleeding was appeared, and HE staining results also proved that the intestinal bleeding in mice and caused damage to the small intestinal barrier. As can we see in Figure 2, the contents of Evans blue increased, which indicated that the intestinal vascular barrier was destroyed. Animal experiments have shown that when the intestine is damaged, it will directly destroy the mucosal function, causing the epithelium of the villi top of the intestinal mucosa to fall off, and the permeability of the intestinal mucosa increases; meanwhile, a large amount of D-lactic acid produced by bacteria in the intestine will enter the blood through the damaged mucosa, raising plasma acid levels [38]. Soluble VE-cadherin is the external functional area of VE-cadherin that has fallen off. The exfoliation of the external functional area of VE-cadherin causes damage to the adhesive connection. These results demonstrated that intestinal vascular barrier function is destroyed, and the contents of Evans blue, D-lactic, and S-VE-cadherin are increased. The model of intestinal injury induced by dasatinib in mice was successfully established.

**Figure 3:**
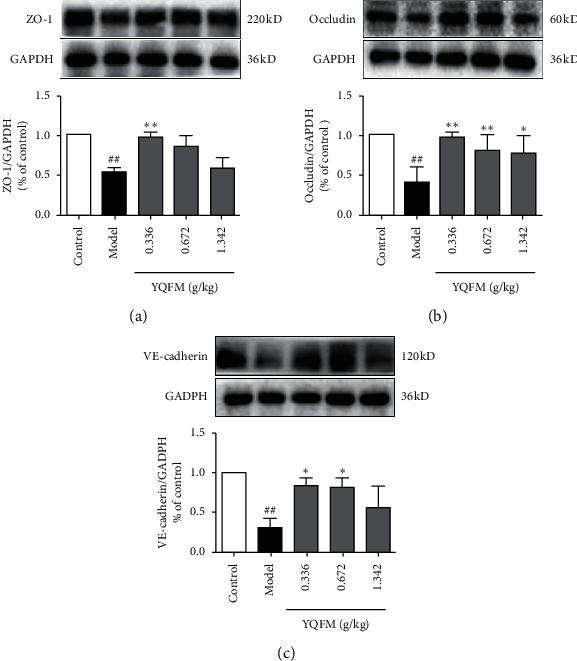
Effect of YQFM on the expression of connexin in mice with Dasatinib. YQFM (0.336, 0.672, 1.342 g/kg, i.p.) was injected intraperitoneally 1 h before being administered with dasatinib (70 mg/kg for 3 d, i.p.). (a–c) Representative western blots and the quantitative analysis of the ratio of ZO-1, occludin, and VE-cadherin. Data are expressed as the mean ± SD, *n* = 3. ^###^*P* < 0.001 vs. control mice; ^##^*P* < 0.01 vs. control mice; ^#^*P* < 0.05 vs. control mice; ^*∗∗∗*^*P* < 0.001 vs. model group; ^*∗∗*^*P* < 0.01 vs. model mice; ^*∗*^*P* < 0.05 vs. model group.

As shown in Figure 3, YQFM was given in advance to protect the intestinal barrier and reduce the degradation of ZO-1, occludin, and VE-cadherin. Tight junctions and adherens junctions play an important role in maintaining the endothelial barrier, and protecting the endothelial barrier has become a new strategy for treating inflammatory bowel disease [39]. Connexin protein between endothelial cells (ECs) is the key to maintain endothelial cell function. The tight junction proteins in EC include ZO-1, claudin-5, occludin, and several JAMs. Previous studies have shown that ZO-1 and occludin protein could be regarded as a characteristic marker of TJ [40, 41], ZO-1 can regulate the formation of blood vessels and maintain the connection tension, and ZO-1 also can binds to F-actin and regulates of cytoskeleton by actomyosin [42]. VE-cadherin is a protein specifically expressed in endothelial cells and plays an important role in maintaining adhesion junctions [43]. There is a close relationship between VE-cadherin and ZO-1.

**Figure 4:**
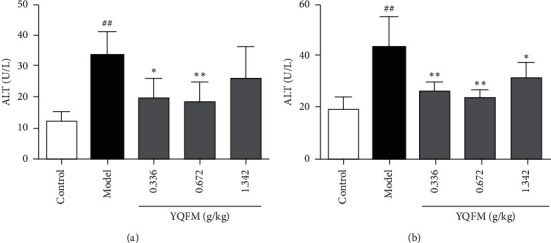
Effect of YQFM on the contents of ALT and AST in mice with Dasatinib. YQFM (0.336, 0.672, 1.342 g/kg, i.p.) was injected intraperitoneally 1 h before being administered with dasatinib (70 mg/kg for 3 d, i.p.). (a, b) Content of ALT and AST were analysed by using kits. Data are expressed as the mean ± SD, *n* = 6. ^##^*P* < 0.01 vs. control mice; ^#^*P* < 0.05 vs. control group; ^*∗∗*^*P* < 0.01 vs. model mice; ^*∗*^*P* < 0.05 vs. model group.

Now, more and more evidence shows that intestinal function is closely related to liver function [44]. When the permeability and integrity of the intestinal wall are affected, a large amount of bacterial products in the intestine enter the liver through the intestinal wall, resulting in impaired liver function. The activity of ALT and AST in the liver is an indicator commonly used to reflect liver injury. As shown in Figure 4, YQFM can significantly reduce the content of ALT and AST in the liver. It showed that YQFM can protect the liver function by reducing the permeability of the intestinal wall.

**Figure 5:**
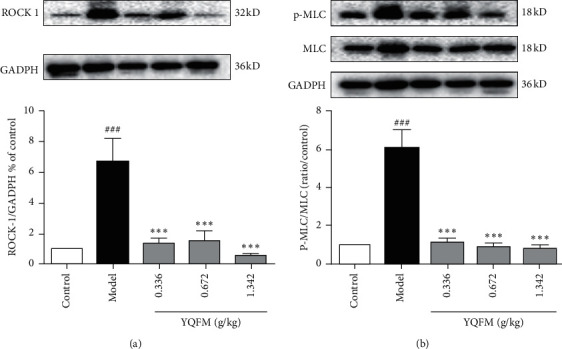
Effect of YQFM on the ROCK-1/MLC signaling pathway in mice with Dasatinib. YQFM (0.336, 0.672, 1.342 g/kg, i.p.) was injected intraperitoneally 1 h before being administered with dasatinib (70 mg/kg for 3 d, i.p.). (a, b) Representative western blots and the quantitative analysis of the ratio of ROCK-1 and P-MLC. Data are expressed as the mean ± SD, *n* = 3. ^###^*P* < 0.001 vs. control group; ^*∗∗∗*^*P* < 0.001 vs. model group.

Dasatinib promotes endothelial cell permeability through the RhoA-ROCK pathway [10]. The ROCK signaling pathway not only regulates the distribution and expression of ZO-1, Occludin, and VE-cadherin but also can accelerate the process of actin aggregation and phosphorylate myosin light chain, let the cytoskeleton (F-actin) contract, further changing the structure of the connexin and its function. So, we investigated how YQFM influenced the ROCK/MLC signaling pathway. As shown in Figure 5, YQFM inhibits the activation of ROCK-1 and phosphorylation of MLC. It is consistent with the efficacy of YQFM previously found, and it can be confirmed that one of the reasons why Dasatinib causes adverse reactions is the activation of the ROCK/MLC signaling pathway, leading to blood vessel leakage.

It is possible that as YQFM has a shorter duration, certain doses of YQFM did not significantly inhibit the degradation of connexin. As it is mentioned above that the function of YOFM did not show a dose-dependent manner, this possible reason is due to the complexity of the components in YQFM. Some components may be present that could inhibit the main components from working, or competitive inhibition might exist between different components in YQFM, so further improvement is needed. Other drugs currently in clinical use, such as nonsteroidal anti-inflammatory drugs (Aspirin), antithrombotic drugs (Dabigatran-etexilate), and some chemotherapy drugs (Methotrexate), have reported gastrointestinal bleeding [31, 45, 46]. The key molecules involved in the bleeding process include VEGF, COX1/2, NF -*κ*B, ROCK, and MLC. Therefore, YQFM may have a potential therapeutic effect on relieving the bleeding caused by the abovementioned drugs, and further research is needed.

In summary, the results of this study provided a new method for improving the side effects of clinical drugs. Preadministering with YQFM restored intestinal blood flow and reduced the degradation of ZO-1, occludin, and VE-cadherin and, meanwhile, reduced the plasma S-VE-cadherin and D-lactate content, protecting the liver function and reducing the content of ALT and AST. It inhibited the activation of ROCK-1 and reduced the phosphorylation of MLC. This study suggested that when applying agents with higher risk of side effects, it may be a feasible approach to combine with protective drugs to reduce the risk of side effects and provides pharmacological evidence for expanding the clinical application of YQFM.

## Data Availability

Data can be obtained from the corresponding author upon request.

## Abbreviations

abrSMS: Sheng-mai San
YQFM: YiQiFuMai powder injection
MLC: Myosin light chain
ROCK: Rho-associated kinase
PVDF: Polyvinylidene fluoride
SDS: Sodium dodecylsulfate
AJs: Adherens junction
TJs: Tight junctions
ALT: Alanine aminotransferase
AST: Aspartate aminotransferase
ZO-1: Zona occludens-1
MLC: Myosin light chains.

## Additional Points

*Highlights*. (i) YQFM reduced vascular leakage and protected the connexins induced by Dasatinib. (ii) YQFM inhibited the levels of ALT and AST induced by Dasatinib. (iii) YQFM modulated the ROCK/MLC signaling pathway induced by Dasatinib.

## Supplementary Materials

Supplementary Materials The supplementary file includes the graphical abstract.
