# Supplementary material for: YQFM Alleviates Side Effects Caused by Dasatinib through the ROCK/MLC Pathway in Mice
Source: Evid Based Complement Alternat Med. 2020 Aug 29;2020:4646029. doi: 10.1155/2020/4646029 (PMC7475753; doi:10.1155/2020/4646029)
Supplement: Supplementary Materials — The supplementary file includes the graphical abstract. [file 4646029.f1.doc]

**Graphical abstract**
